# Supplementary material for: Key Indications for Passive Immune Prophylaxis Against SARS-CoV-2 Infection in Malignant Hematological Disorders: An Analytic Hierarchy Process by an Ad Hoc Italian Expert Panel
Source: Vaccines (Basel). 2025 Dec 30;14(1):46. doi: 10.3390/vaccines14010046 (PMC12846593; doi:10.3390/vaccines14010046)
Supplement: Supplementary file 1 [file vaccines-14-00046-s001.zip › vaccines-4011378-supplementary.pdf]

Supplementary Table S1A. Host-related risk factors of SARS-CoV-2 infection and outcomes: systematic review of high-quality evidence.

Risk factor	Outcomes	Meta-analyses	Other references
Age	✓ Age inversely correlated with serological response to active immune prophylaxis in cancer patients [OR 1.29 (1.11-1.50)]<br>✓ Any additional age year increased the risk of severe COVID19 (OR 1.05)<br>✓ COVID19 fatality rate increased from 24% in HM patients aged 65-70 years to 41% in those aged over 80 years in a large cohort of 3603 patients (EPICOVIDEHA): at multivariate analysis, age over 80 years reported an HR for death of 2.5 , age 75 and HR of<br>✓ Age (over 50 or over 60 years) also contributed to higher rate of severe infection in HSCT patients<br>✓ Age over 65 years was associated with worse hospitalization outcomes: hospital mortality being twice as high (HR 2.0 (1.1-5.2), p =0.05)<br>✓ Age over 70 worsen infection outcomes in immunocompromised individuals	Yang 2022<br>Noori 2022<br>Randi 2023<br>Abodunrin 2025<br>Alinaghi 2022	Zhu 2023,<br>Linton 2024 ASH<br>Polyakov 2022<br>Rossi 2023<br>Anand 2024<br>Salamanton-Garcia 2024
Male gender	Male gender was associated with a lower rate of seroconversion after vaccination in cancer patients OR 1.34(1.13e1.58-	Yang 2022	
Comorbidity	✓ ≥2 comorbidities (OR, 3.47; 95% CI, 1.63–7.64; p = 0.001) was associated with a higher risk of pneumonia in ITP patients<br>✓ Each co-morbidity was associated with an additional risk of severe COVID19 in veterans with HM diseases (OR per comorbidity, 1.35; 95% CI, 1.29-1.43)<br>✓ Specific co-morbidities such as diabetes mellitus, coronary artery disease, end-stage renal failure, prior respiratory infection worsen infection outcomes in immunocompromised individuals<br>✓ Diabetes in particular independently predicted mortality in HM patients hospitalized for COVID19 (HR 2.47 (0.94–6.48))<br>✓ Chronic cardiopathy, renal impairment and liver disease significantly increased COVID19-related mortality at multivariate analysis of 2603 elderly HM patients (EPICOVIDEHA) but the HR were lower than 1.6<br>✓ In the general elderly population, dementia and malnutrition were the most powerful predictors of adverse COVID19 outcomes (OR higher than 2.5) but COPD and diabetes were also significantly correlated with the outcome.<br>✓ Renal failure was a specific comorbidity independently predictive the outcome fo COVID19 infection in MM patients in the EPICOVIDHEA large international cohort	Alinaghi 2022<br>Abodunrin 2025<br>Naimi 2022	Zhu 2023 Wu 2024<br>Zhu 2024<br>Musto 2024<br>Stahl 2021
Severe hypogammaglobulinemia	✓ IgG, IgA and IgM serum levels correlated to a higher seroconversion rate after vaccination (OR of missed seroconversion about 0.40)<br>✓ Prophylactic immunoglobulin reduced the risk of clinically documented	Yang 2022	

	✓ infection (CDI) by 28% (n = 2 trials; RR, 0.72; 95% CI, 0.54-0.96), and vaccination reduced the risk by 63% (RR, 0.37; 95% CI, 0.30-0.45).		
Severe lymphopenia	✓ High neutrophil to lymphocyte ratio at infection in healthy individuals and in HM patients has been associated with a worse infection outcome.<br>✓ Lymphopenia independently predicted 60-day mortality in HM patients hospitalized for COVID19 (HR 4.77 (1.45–15.72)<br>✓ Lymphopenia below 500/mcl independently increased COVID19-related mortality (HR 1.65) in elderly HM patients	Sarkar 2022<br>Meejun 2023<br>Zinellu 2022<br>Li 2021<br>Patharashati 2022	Bozkurt 2024<br>Wu 2024<br>Rossi 2023<br>Themlaoui 2025
Neutropenia	✓ A case-control study matched 130 HM patients (mostly AML) with grade 4 neutropenia to control HM patients without severe neutropenia.<br>✓ Sepsis developed in half of the patients and ICU transfer was required in 60%.<br>✓ Overall mortality rate ranged from 0% in non-ICU patients younger than 60 years to over 80% in ICU admitted patients and in those older than 60 years.<br>✓ Mortality was significantly higher than the reference group without severe neutropenia: 48-52% vs 14-37%<br>✓ Neutropenia (ANC below 500) was an independent predictor of bad COVID19 outcome in MM patients (EPICOVIDEHA)<br>✓ In a separate case series of 335 lymphoma patients, hospital mortality (21% overall) was independently predicted by grade 4 neutropenia (HR 1.5 (0.9-2.7), p =0.09<br>✓ Febrile neutropenia was associated to poor outcomes of COVID19 infection: 8411 hospital admissions for febrile neutropenia and positive COVID19 test were compared with those with a negative COVID19 test: all cause mortality was 10.8% in COVID19 positive patients vs 5.4% in negative ones (OR 2.12, 95% CI 1.89-2.38,p=0.000), possibly due to a higher risk of cardiac complications (sudden cardiac arrest 19% vs 13%)<br>✓ Neutrophil count did not predict COVID19related mortality in a large cohort of elderly HM patients reported by EPICOVIDEHA		Kaluzylaia 2022<br>Polyakov 2022<br>Rossi 2023<br>Musto 2024<br>Stahl 2021
Prior splenectomy	✓ In a cohort of 191 splenectomized ITP patients 76% response rate to SARSCoV2 vaccination was reported.<br>✓ During COVID19 infection vaccinated patients showed a lower risk of severe infections (odds ratio [OR], 0.13; 95% confidence interval [CI]: 0.05–0.36; p < 0.001), hospitalization (OR, 0.13; 95% CI, 0.04–0.48; p = 0.002), and ITP exacerbation (OR, 0.16; 95% CI, 0.04–0.67; p = 0.012).<br>✓ Previous splenectomy (OR, 1.98; 95% CI, 1.09–3.61; p = 0.03) was associated with platelet count decline after COVID19 infection in ITP patients.<br>✓ In a Danish case-control study of 552 splenectomized individuals did not report higher irks of COVID19 infection (adjusted OR: 0.89; 95% CI: 0.73-1.08), however, infected patients had an increased risk of hospitalization or death (adjusted OR for combined endpoint: 1.44; 95% CI: 0.79–2.61).		Riviere 2024 BJH<br>Bianchi 2023 BJH<br>Liu 2024<br>Bojesen 2021

	✓ In an Apulian study, the infection rate was 15/100 PY in 1219 splenectomized inhabitants (64% vaccine coverage, 15% booster dose) with a proportion of re-infection equal to 6.4%; the proportion of hospitalization was 2.9%, with a case-fatality rate of 2.6%.		

Supplementary Table S1B. Disease-related risk factors of SARS-CoV2 infection and outcomes: systematic review of high-quality evidence. Lymphoproliferative diseases.

**Risk factor**	**Outcomes**	**References: meta-analyses**	**References: not meta-analysis**
MM/SMM	✓ COVID19-related hospitalization rate in MM patients was 53% (95% CI: 40.81, 65.93), ICU admission rate was 17% (95% CI: 11.74, 21.37) and mortality rate 22% (95% CI: 15.33, 28.93).<br>✓ Seroconversion after complete vaccination in MM was 76-78%, that is a risk ratio of 0.79 (0.75–0.83) versus healthy recipients.<br>✓ Seroconversion risk ratio in SMM (treatment naiive pts) was not significantly worse than healthy population risk ratio 0.96 (0.75-1.24).<br>✓ Maintenance lenalidomide impairs seroconversion but T-cell response might be still adequate.<br>✓ Immunosuppressive drugs alone or in combination with corticosteroids increased the rate of severe COVID19 in HM assisted by the VA (OR, 2.32; 95% CI, 1.93-2.80)	Harandi 2024,<br>Mahmud 2024<br>Chuleeranux 2022<br>Uaprasert 2022<br>Ito 2022<br>Gagelman 2022	Beer 2024<br>Salamanton-Garcia 2024<br>Anand 2024
CLL	✓ Pooled seroconversion rate after 2 doses (or complete vaccination) was 47-54%. The risk ratio of seroconversion after 2nd dose was 0.51 (0.42–0.62) as compared to healthy recipients.<br>✓ However, treatment-naiive patients did not show a significantly impaired response (risk ratio 0.79 ns). IGVH mutational status was associated with OR of missed seroconversion 0.52 (0.28-0.98)<br>✓ Long viral shedding was reported in CLL patients, despite antiviral therapies.	Akbarzadeh 2024<br>Noori 2022,<br>Yang 2022,<br>Uaprasert 2022,<br>Ito 2022,<br>Gagelman 2022	Minoia 2022
NHL	✓ Pooled seroconversion rate after complete vaccination was 58-68%, which is significantly lower than healthy individuals (risk ratio 0.54-0.60).<br>✓ Similar rates were reported in indolent and aggressive NHL, but heterogeneous data were reported for indolent untreated NHL.<br>✓ In some analyses, seroconversion risk ratio in indolent untreated NHL (naiive) was similar to healthy population (RR 0.90, 95% CI 0.81-1.01), while in others its was lower than in aggressive NHL.<br>✓ Long antiviral shedding despite antiviral therapies	Noori 2022<br>Uaprasert 2022<br>Ito 2022<br>Gagelmann 2022	Minoia 2022<br>Maruyama 2024, 2025<br>Perry 2021<br>Lim 2024
HL	✓ Seroconversion rate was 80-91% not statistically significantly lower than healthy population (RR 0.94 95% CI 0.83-1.06)	Uaprasert 2022<br>Ito 2022<br>Gagelmann 2022	

Supplementary Table S1C. Disease-related risk factors of SARS-CoV2 infection and outcomes: systematic review of high-quality evidence. Myeloid neoplasms and non-neoplastic disorders.

**Risk factor**	**Outcomes**	**References: meta-analyses**	**References: not meta-analysis**
AML	✓ Myeloid neoplasms showed a relative risk of seroconversion after 2nd vaccine dose versus healthy individuals of 0.81 (0.72–0.91)<br>✓ Seroconversion rate after full vaccination in AML was 88% but mortality rate in infected AML patients was high twice as high as compared to MM, CML, MPN and CLL.<br>✓ Out of 388 AML patients reported by EPICOVIDEHA international survey from Jan 2020 to September 2021 (the vast majority exposed or recently exposed to treatment) the majority incurred severe or critical COVID19 infection (62%), therefore the infection required chemotherapy schedule modification in 44.8%, treatment delay in 17% and treatment discontinuation in 27% with overall mortality of 46%: in half of the cases death was directly attributable to COVID19.<br>✓ Mortality rate after COVID19 was also reported in 3474 AML patients across 60 US institutions (Sood 2024 ASH) and ranged from 24 to 41%	Uaprasert 2022<br>Noori 2022	Rossi 2023<br>Marchesi 2023<br>Sood 2024<br>Sivasubramian 2024<br>Mulanovich 2022
MDS	✓ Seroconversion rate after complete vaccination was 83.7%<br>✓ The risk ratio of seroconversion after 1st vaccine dose was 0.48 (0.27–0.86) and after 2nd vaccine dose was 0.77 (0.50–1.18) in overall MDS patients compared to healthy recipients	Noori 2022<br>Uaprasert 2022	Salamanton-Garcia 2024
MPN	✓ Seroconversion rate after vaccination was 83-87%. The risk ratio of seroconversion after second vaccine dose was 0.85 (0.79–0.91) as compared to healthy individuals	Noori 2022<br>Uaprasert 2022<br>Gagelman 2022	
CML	✓ No significant increased risk of infection or reduced risk of seroconversion after vaccinations (confirmed by ad-hoc prospective studies: Galvis 2022)<br>✓ Overall COVID19 associated mortality during initial pandemic wave was 5.5% in CML patients (Breccia)<br><br>✓ The iCMLf CANDID study collected data on SARS-CoV-2 positive CML pts from 157 institutions in 49 countries until November 2021 and collected 1050 pts (79% in chronic phase with major molecular response): mortality rate was 6% but declined to 4% in patients with a complete cytogenetic response vs 13%.<br>✓ Severe COVID19 occurred more frequently (39%) of patients with accelerated or blast phase disease and in chronic phase patients without major molecular response (10% vs 19% p<0.0001).	Ali 2023	Breccia 2022,<br>Rotetrdam 2022<br>Galvis 2022<br>Radich 2022<br>Graf 2023<br>A-Ashwah 2024

	✓ The mortality rate for MMR, no MMR and AP/BC was 4%, 11% and 26% respectively. By multivariate analysis no MMR, AP/BC, lower country income, older age and coexisting comorbidities were independent risk factors for mortality.<br>✓ The iCMLf CANDID study also addressed 95 pts were in TFR at the time point of SARS-CoV-2 infection of which 89 (93.68%) recovered and 6 deceased (6.32%). Median age of TFR pts was 57 years, male were 51 (53.68%)..69 pts remained in TFR (93%) and 5 pts lost TFR. Additionally, there were no statistically differences in hospitalization rate (16% vs 23%, p=0.12) and severity of COVID-19 symptoms (12.6% vs 12%, p=0.87) comparing TFR and TKI treated pts.		
Immune cytopenias	✓ About 30% of ITP patients experienced a flare after the diagnosis of COVID-19; in a large Chinese prospective cohort 18% showed decline of platelet count after infection and 4% develop overt pneumonia. Risk factors for a decrease in the PLT count included baseline PLT count <50 × 10^9/L (OR, 1.76; 95% CI, 1.25–2.46; p = 0.001), maintenance therapy including thrombopoietin receptor agonists (TPO-RAs) (OR, 2.27; 95% CI, 1.60–3.21; p < 0.001) and previous splenectomy (OR, 1.98; 95% CI, 1.09–3.61; p = 0.03). Risk factors for pneumonia included age ≥40 years (OR, 2.45; 95% CI, 1.12–5.33; p = 0.02), ≥2 comorbidities (OR, 3.47; 95% CI, 1.63–7.64; p = 0.001), maintenance therapy including TPO-RAs (OR, 2.14; 95% CI, 1.17–3.91; p = 0.01) and immunosuppressants (OR, 3.05; 95% CI, 1.17–7.91; p = 0.02)	Ono 2024<br>Chue 2024	Fu 2024<br>Chen 2024
Bone marrow failure syndromes	✓ A few data are reported on aplastic anemia patients developing COVID19 infection		Pike 2023<br>Zhao 2023
Hemoglobinopathies	✓ Both sickle cell trait and sickle cell disease were associated to an increased risk of COVID19-related death (OR 1.43 and OR 1.94) as compared to control individuals. Albo beta-thalassemia major patients face a higher irks of COVID19-related death (RR 1.85).	Dawudi 2024<br>Liang 2023<br>Michelon 2023<br>Borborema 2023	

Supplementary Table S1D. Therapy-related risk factors of SARS-CoV2 infection and outcomes: systematic review of high-quality evidence

**Risk factor**	**Outcomes**	**References: meta-analyses**	**References: not meta-analysis**
Response	✓ Higher seroconversion after vaccination in responders (vs active disease: 35%)	Gagelmann 2022	Rossi 2023
HSCT	✓ Seroconversion rate was 76-82% (81% in HCT) Risk ratio of seroconversion after 2nd vaccine dose vs healthy individuals 1.15 (1.05–1.26). Allogeneic vs autologous 0.97 (0.86–1.09). However if less than 24 months elapsed between vaccination and HCT the risk ratio was 0.75 (0.61–0.93)<br>✓ Pooled mortality associated with COVID19 infection in HCT recipients was 17% (95% CI 13-22%) and HR 9.04 (2.63–31.04)<br>✓ NRM does not seem to be affected by prior COVID19 infection but a relevant HSCT delay might be caused by peritransplant infection	Demel 2024, Randi 2024 Noori 2022<br>Uaprasert 2022<br>Gagelmann 2022<br>Randi 2024	Ahmad 2024 ASH (abstr 3554)<br>Wu 2024<br>Siniaev 2021, 2022, 2024<br>Mansour 2023<br>Barnes 2023<br>Attolico 2022
CAR-T	✓ Seroconversion rate was very low: 18-42%	Demel 2024,<br>Uapraset 2022<br>Wang 2022<br>Gagelmann 2022	Zheng 2024 ASH (5170a)<br>Barnes 2023
ASCT (NHL, MM)	✓ Seroconversion rate higher than 80%<br>✓ Pooled mortality associated with COVID19 infection was 14% (95% CI 8-22%)	Wang 2022 Uaprasert 2022 Randi 2023,<br>Gagelmann 2022	Barnes 2023
Bispecific antibodies	✓ A post-hoc analysis of MajesTEC-1 trial focused on 165 MM patients (13 vaccinated before teclistamab, 99 ones after teclistamab) treated with teclistamab: COVID19+ was reported in 29% with grade 3-4 infection in 21.2% of the patients and deaths in 10.9% in a median of 22.8 months (18 deaths related to COVID19). Teclistamab was interrupted in 60% of COVID19+ patients. COVID19 infection impacted on PFS and OS.<br>✓ IGIV seemed to limit the rate of severe COVID19 in MM recipients of bispecific antibodies in the real life Australian series.<br>✓ Out of 42 NHL patients treated with glofitamab in the real world, 9 ones (21%) got the infection and 5 ones (12%) died due to COVID19.<br>✓ Similarly 8 out of 108 patients treated with mosunetuzumab and polatuzumab died for COVDI19 pneumonia.	Reynolds 2023	Van de Donk 2024<br>Nooka 2024<br>Fares 2023<br>Kyvsgaard 2023<br>Ferhanoglu 2022<br>Olzewski 2023<br>Lim 2023

Anti CD20 or CD19 Moab	✓ OR 2.95; 95% CI 2.30-3.78<br>✓ OR 2.14; 95% CI 1.37-3.35<br>✓ Seroconversion rate was low: 31-36%, which is a risk ratio of 0.45-0.47 versus healthy individuals<br>✓ chemoimmunotherapy [odds ratio (OR), 3_42; 95% confidence interval (CI), 1_04–11_21; P = 0_04] was associated with a lower rate of seroconversion: this effect did not decline after 180 days.<br>✓ However it was specifically dependent on timing between exposure and vaccination (15-23% vs 63% recent vs late) with increasing work in the time frames within 6 mo from exposure, within 12 mo.<br><br>✓ The risk was still persistent after 12 mo from exposure (risk ratio 0.60; 0.52-0.68).<br>✓ Risk ratio is particularly low 0.20 (0.11–0.37) ((OR of missed seroconversion 2.72(1.28-5.78))<br>✓ if less than 12 mo elapsed between treatment and vaccination<br>✓ When oral antivirals and monoclonal agents were available for outpatients, exposure to anti CD20 was still a predictor of hospitalization for COVID19 predictors of hospitalization were: recent administration of anti-CD20 (aOR=13.03, 95%CI=1.86-91.3),<br>✓ Tafasitamab add-on therapy significantly increased the rate of COVID19 infection and pneumonia (5% vs 1%) in combination with lenalidomide in the inMIND study	Kow 2023<br>Demel 2024,<br>Uaprasert 2022<br>Wang 2022,<br>Tang 2022,<br>Noori 2022,<br>Ito 2022,<br>Yang 2022,<br>Gaglemann 2022	Minoia 2022<br>Passamonti 2020
BTKi or BCL2i	✓ Pooled seroconversion rates after vaccination in BTKi treated patients ranged from 23% to 36.6% (risk ratio 0.49; 0.37-0.64)<br>✓ Seroconversion rates in BCL2i treated individuals was 26-39% (most of venetoclax-exposed patients being CLL and not AML)<br>✓ Continuous BTK inhibitors was hypothesized to be protective effect on the outcome of COVID-19 infection (OR 0.44, p = 0.043) in the indolent JCLG lymphoma cohort	Wang 2022,<br>Tang 2022<br>Uaprasert 2022<br>Ito 2022<br>Gaglemann 2022	Zhu 2024
AML (or hr MDS) induction therapy	✓ Seroconversion rate in patients undergoing chemotherapy for blood cancers was 75.1%; 95% CI, 63.6–83.9% vs 80.4%; 95% CI, 73.7–85.8%, in not treated patients	Uaprasert 2022	Marchesi 2023
JAKi	✓ Seroconversion rate in JAKi treated individuals was 42-63% and ruxolitinib risk ratio of seroconversion after 2nd vaccine dose 0.73 (0.57–0.94). Patients currently exposed to JAKi had significantly lower seroconversion rates (64.2%; 95% CI, 53.1–74.0%, $I^2 = 0\%$) than those unexposed to JAKi (90.4%; 95% CI, 84.9–94.1%, $I^2 = 0\%$), ($P < 0.001$).	Uaprasert 2022,<br>Wang 2022<br>Noori 2022<br>Gaglemann 2022	
TKI	✓ Seroconversion rate was higher than in not-treated HM patients 93.9%; 95% CI, 80.2–98.3	Uaprasert 2022	
IMIDs	✓ Seroconversion rate was 80.6%; 95% CI, 67.6–89.2% (similar to not-treated HM patients) and risk ratio of seroconversion after 2nd vaccination 0.97 (0.87–1.08)	Noori 2022,<br>Uaprasert 2022	
Lr MDS on therapy (IMIDs or IS)	✓ No specific data on disease plus therapy combination subgroups		Beer 2024

Igiv prophylaxis	✓ The use of IVIG indicated a significant reduction in the odds of mortality (pooled OR = 0.69; 95% CI 0.50–0.96) relative to nonuse of IVIG. Subgroup analysis in patients with a severe course of COVID-19 revealed no significant reduction in the odds of mortality (pooled OR = 0.58; 95% CI 0.29–1.16).<br>✓ In a study of 125 elderly patients with DLBCL undergoing reduced intensity R-CHOP therapy, 89 patients presented with hypogammaglobulinemia at diagnosis, and 56 patients received IGIV. IVIG administration remarkably reduced COVID-19 infection rates compared to non-IVIG recipients (8.9% vs. 24.6%; p =0.040).<br>✓ IGIV limited to 12% the rate of COVID19 infection in 52 MM patients treated with monoclonal or bispecific antibodies in the real life. No infection-related death was reported.<br>✓ Prophylactic immunoglobulin reduced the risk of clinically documented<br>✓ infection (CDI) by 28% (n = 2 trials; RR, 0.72; 95% CI, 0.54-0.96), and vaccination reduced the risk by 63% (RR, 0.37; 95% CI, 0.30-0.45).	Ramachandran 2024<br>Fatemi 2023<br>Chai 2021	Baek 2024<br>Lim 2024<br>Dimbleby 2025
Anti CD38	✓ Seroconversion rate was 81.4%; 95% CI, 63.2–91.7%,<br>✓ Risk ratio of seroconversion after 2nd vaccination was estimated to be 0.89 (0.76–1.05) and 0.86 (0.76-0.96) in 2 different meta-analyses	Noori 2022<br>Uaprasert 2022<br>Ito 2022	Farina 2024
Proteasome inhibitors	✓ Seroconversion rate was 83.1%; 95% CI, 59.8–94.2%,<br>✓ Risk ratio of seroconversion after 2nd vaccination 1.05 (0.95–1.14)	Noori 2022<br>Uaprasert 2022	

Supplementary Table S2. Systematic review of the effect of TC PrEP in immunocompromised patients: meta-analyses.

Study	Soeroto, 2023	Wang, 2023	Suribhatla, 2023
Number of included studies	6	5	18
Number of pooled patients assigned to TC	6966	7295	23,345 (5,438*)
Infection rate	OR: 0.24; 95% CI: 0.15–0.40, $p < 0.00001$	RR 0.28, p<0.01	40.54%^
Hospital admission	OR: 0.13; 95% CI: 0.07–0.24, $p < 0.00001$		66.19%
Severe COVID19	OR: 0.13; 95% CI: 0.07–0.24, $p < 0.00001$		82.13% (ICU)
Fatal COVID19	OR: 0.17; 95% CI: 0.03–0.99, $p = 0.05$	RR 0.5, p<0.01	92.39%
Severe adverse events		OR 0.9, p=0.048	

^prevented breakthrough infections * immunodepression related to hematologic disorders

Supplementary Table S3. Systematic review of PrEP with T/C in patients with HM.

Author, year	Country	Time of the study	Type of study	N. pf pts treated	breakthrough COVID19 cases, %	Outcome of breakthrough COVID-19 cases		
						Hospitalizations %	ICU admissions, %	Deaths, %
Stuver, 2022	US	2021 (Q3-4)	P, SC	52	3.8	0	0	0
Al-Obaidi, 2023	US	2022 (Q1-2)	R, SC	374	6.1	16.7	0	0
Mauro, 2022	IT	2022 (Q1-3)	R, MC	89	28.1	20	0	0
Davis, 2022	US	2022 (Q1-3)	R, SC	251	10.8	14.8	0	0
Duminuco, 2023	IT	na	R, SC	198	22.2	4.5	0	0
Duminuco, 2023	IT	na	R, SC	40	22.5	0	0	0
Hall, 2023	AU	2022 (Q3-4)	P, SC	93	15.1	7.1	0	0
Jondreville, 2022	France	2021 (Q4) - 2022 (Q1-2)	R, MC	161	13.7	0	0	0
Xue, 2023	IT	2022 (Q2-4)	R, SC	57	22.8	7.7	0	0
Thurlapati, 2023	US	2022 (Q1-3)	R, SC	126	10.3	23.1	0	0
Chang, 2023	US	2022 (Q1-4)	P, SC	28	14.3	0	0	0
Moon, 2023	US	2022 (Q1-2)	R, SC	958	na	na	0	0
Kamboj, 2023	US	2022 (Q4) 2023 (Q1)	R, SC	na	na	21.2	na	3.8
Zerbit, 2022	France	2021 (Q4) - 2022 (Q1)	P, SC	102	4.9	20	20	20
Kertes, 2022	Israel	2021 (Q4) - 2022 (Q1-2)	R, SC	164	7.9	7.7	0	0
Laracy, 2023	US	2022 (Q1-2)	R, SC	887	10.9	8.2	1	1
Nguyen, 2022	France	2021 (Q4) - 2022 (Q1)	P, MC	306	3,9	8.3	na	8.3
Marchesi, 2023	EU	na	R, MC	NR	na	21.3	6.4	4.2
Tatetsu, 2023	Japan	2022 (Q4) 2023 (Q1)	R, SC	67	7.4	0	0	0
Callegari, 2023	IT	2022 (Q2-3)	P, SC	93	20.4	26.3	na	21.0
Hijano, 2023	US	2021 (Q4) 2023 (Q1)	P, SC	27	29.2	14.3	0	0
Ntanasis-Stathopoulos, 2023	Greece	2022 (Q1-4) 2023 (Q1-2)	P, SC	111	8.1	0	0	0

Zamprogna, 2023	IT	2022 (Q2-3)	R, MC	139	5.1	14.3	0	4.8
Galli 2023	IT	2022 (Q2-4) 2023 (Q1)	R, MC	24	58.3	na	na	0
Demel 2024	Czech Republic	2022 (Q2-3)	R, MC	606	15	25.0	20.1	30.2
Trepl 2024	Switzerland	2022 (Q1-4) 2023 (Q1-2)	R, SC	40	15	0	0	0
Reimann 2024	Austria	2022 (Q2-3)	R, MC	155	25.8	5	5	5
Laracy 2023	US	2022 (Q1-2)	R, SC	892	10.9	8.2	na	1
Lee 2024	Korea	2022 (Q3)	P, SC	94	13.8	0	0	0
Guarnera 2024	IT	2022 (Q1-4)	R, SC	53	38	20	na	0
Benjiamini 2024	Israel	2022 (Q1-4)	P, SC	70	29	15	0	0
Braitsch 2024	Germany	2022 (Q2-4)	R, SC	54	32	37.5	12.5	12.5
Azuly 2024	US, Brazil, Spain, Turkey	2022 (Q1-2)	R, MC	215	11.2	12.5	12.5	4.2
Fraczkiewicz 2024	Poland	2022 (Q1-4)	R, MC	69	8.7	0	0	0
Haraguki 2023	Japan	2022 (Q4) 2023 (Q1)	R, SC	257	7.5	66.7	0	0
Rigolin 2024 ASH	IT	2022 (Q2-3)	R, MC	512	31	na	0.8	0.8
Angotzi, 2023	IT	2022 (Q2, Q3)	P, SC	35	20	2.9	0	0
Azuly, 2025	Israel	2022 (Q2,Q3)	R, MC	215	11.2	na	11.5	4.2
Total				9660	13.1 [a]	14.9 [b]	2.6 [c]	3.4 [d]

a: 1138 of 8702 evaluable cases; b: 132 of 883 evaluable cases; c: 21 of 813 evaluable cases; 36 of 1067 evaluable cases

Legend to the table. Legend: US = United States of America, AU = Australia, IT = Italy, EU = European Union. R = retrospective, P = prospective (observational), MC = multicenter, SC = single center

Supplementary Table S4. Host-, disease-, therapy-related risk factors: relevancy scores and subgroups.

Domain	Risk factor	Mean score	Mean / SD ratio	Operationalization
Therapy	**Candidates to cellular therapies:**[1]			
	HSCT[2]	4.6-4.7	6.6-9.7	Allogeneic HSCT planned in 3 months
	CAR-T	4.1-4.6	3.7-6.6	CAR-T therapy planned in 2 months for lymphoma or LLA
	Autologous SCT	3.9-4.4	3.7-5.2	Autologous SCT planned in 3 months for lymphoma or MM
	**After completion of cellular therapies:**			
	After CAR-T[3]	3.8-4.2	2.9-3.7	6 months after CAR-T therapy
	After HSCT[4]	3.9-4.5	3.5-4.6	12 months after allogeneic HSCT therapy
	**Ongoing therapies:**			
	Anti CD20 or CD19 Moab[5]	3.8	3.3	Lymphoma patients ongoing or recently exposed (<6 months) to Moabs
	BTKi or BCL2i[6]	3.3-4.2	3.2-5.1	CLL or lymphoma patients ongoing target therapies
	Chemoimmunotherapy[7]	3.7-3.9	3.2-3.5	ongoing or recently exposed (< 12 months before) c
	Intensive chemotherapy	3.5-3.9	2.1-3.3	Intensive induction (or recue) chemotherapy for AML or high-risk MDS patients
	JAKi	3.5	2.4	Ongoing ruxolitinib, fedratinib, momelotinib or pacritinib
	TKI	2.6	1.7	Ongoing TKI (e.g. imatinib) irrespectively of the TKI generation and dose or therapy duration
	Anti-MM therapies [8]	3.1-4.0	2.0-3.2	IMIDs, anti CD38 and anti BCMA Moabs, proteasome inhibitors
	Immunosuppressive therapy for MDS	3.1-3.3	2.1-2.3	IMIDs or other immunosuppressive agents (e.g. prednisone > 2 mg/Kg for > 2 weeks) for low-risk MDS
	IGIV prophylaxis[9]	3.7	3.5	Anti-infective supportive therapy for CLL patients
	BITE	3.9-4.2	3.7-4.3	Lymphoma and MM patients candidate to receive (or being treated with) BITE therapy
Disease	**Lymphoid neoplasms**:	2.2-2.7	2.0-2.3	On-therapy patients achieved the highest scores
	**Myeloid neoplasms:**			

	AML or hr MDS on therapy	3.5-3.9	2.1-3.3	Therapy included intensive chemotherapy, venetoclax-based chemotherapy and demethylating agents
	MDS lr[10]	2.7-3.1	1.9	On or off immunosuppressive therapies
	MPN on therapy	2.8-3.0	1.6-1.7	MPN patients on cytoreductive therapy
HOST	Relevant comorbidity was operationalized according to WHO, namely including obesity, diabetes and/or chronic conditions including chronic obstructive pulmonary disease, kidney or liver disease, active cancer, and/or disability<br>Severe immunosuppression was operationalized by severe reduction of serum immunoglobulin G levels (below 5 g/l) or severe lymphopenia (below 500/mcl)<br>Overall risk classes including neutrophil count below 1000/mcl or severe immunosuppression were assigned a mildly higher score.<br>Time elapsed since last SARS-CoV-2 vaccination was never responsible for a relevant increase of the estimated risk score.<br>Old age threshold was set at 60 years based on the multivariate analyses performed in most of the studies devoted to hematologic neoplasms.			

Legend: SD = standard deviation, lr = low risk, hr = high risk, IS = immunosuppressive therapy, MM = multiple myeloma, SMM = smouldering myeloma, CLL = chronic lymphocytic leukemia, HSCT = hematopoietic stem cell transplant, BITE = bi-specific antibodies, ASCT = autologous stem cell transplant

Notes to the legend:

1.higher scores were assigned to patients with an older age, comorbidities, recurrent infections, severe immunodepression (IgG<5 g/l or lymphocyte count <500/mcl), recent exposure to anti-CD20 or anti-CD19 Moab, or long time elapsed since last vaccine dose were assigned higher scores. 2. higher scores were assigned to AML patients candidate to HCT after achieving a second complete remission. 3. Significantly lower scores for LLA. 4. higher scores were assigned to patients older than 60 years, with lower lymphocyte counts (<500/mcl) or lower neutrophil counts (<1000/mcl). 5. Ongoing or recently exposed to Moabs 6. higher scores were assigned to patients with an older age, comorbidities, recurrent infections, severe immunodepression (Ig<5 g/l or lymphocyte count <500/mcl), recent exposure to anti-CD20 or anti-CD19 Moab, or long time elapsed since last vaccine dose were assigned higher scores. 7. Older than 60 years or severely immunodepressed (IgG<5 g/l or lymphocyte count <500/mcl). 8. Higher scores were assigned to patients with recurrent infections, relevant comorbidities or severe humoral immune-depression (IgG<5 g/l). 9. 55% of the panelists deemed that different priorization to passive immunoprophylaxis based on IGIV depends on the clinical scenario. Despite ongoing target therapy, CLL patients with severe hypogammaglobulinemia receiving Igiv were scored lower than the same patients not receiving igiv.

Supplementary Table S5. Original 80 risk classes

N	RISK CLASS	N	RISK CLASS
1	Lymphoma patient candidate to autologous SCT	44	CLL patient on BCL2-inhibitor therapy and IGIV
2	Lymphoma patient aged >60 years candidate to autologous SCT	45	CLL patient on BCL2-inhibitor therapy and not on IGIV
3	Lymphoma patient with relevant comorbidities who is candidate to autologous SCT	46	CLL patient recently (< 12 months) exposed to anti-CD20 monoclonal antibodies and receiving IGIV
4	Lymphoma patient candidate to autologous SCT & >12 months elapsed from last anti-SARS-CoV-2 vaccine	47	CLL patient recently (< 12 months) exposed to anti-CD20 monoclonal antibodies and not receiving IGIV
5	Lymphoma patient with severe immunodepression (serum immunoglobulins < 5 g/l and/or absolute lymphocyte count < 500/mcl) who is candidate to autologous SCT	48	CLL patient who is off-treatment > 12 months and on IGIV
6	MM patient candidate to autologous SCT	49	CLL patient who is off-treatment > 12 months and not on IGIV
7	MM patient aged >60 years candidate to autologous SCT	50	Lymphoma patient on chemoimmunotherapy
8	MM patient with relevant comorbidities who is candidate to autologous SCT	51	Lymphoma patient on BKT-inhibitor therapy
9	MM patient candidate to autologous SCT & >12 months elapsed from last anti-SARS-CoV-2 vaccine	52	Lymphoma patient on BCL2-inhibitor therapy
10	MM patient with severe immunodepression (serum immunoglobulins < 5 g/l and/or absolute lymphocyte count < 500/mcl) who is candidate to autologous SCT	53	Lymphoma patient on IMID therapy
11	Lymphoma patient soon (< 3 months) after autologous SCT	54	Lymphoma patients receiving or having recently received (< 12 months) anti-CD20 or anti-CD19 monoclonal agents
12	MM patients soon after (< 3 months) after autologous SCT	55	Lymphoma patients aged > 60 years on chemoimmunotherapy
13	Lymphoma patient candidate to CAR-T therapy	56	Severily immunodepressed lymphoma patients (serum immunoglobulin levels < 5 g/l or absolute lymphocyte count < 500/mcl) on chemoimmunotherapy
14	Lymphoma patient aged >60 years candidate to CAR-T therapy	57	Lymphoma patients aged > 60 years on target therapy
15	Lymphoma patient with relevant comorbidities who is candidate to CAR-T therapy	58	Severily immunodepressed lymphoma patients (serum immunoglobulin levels < 5 g/l or absolute lymphocyte count < 500/mcl) on target therapy
16	Lymphoma patient candidate to CAR-T therapy & >12 months elapsed from last anti-SARS-CoV-2 vaccine	59	Patient with smoldering MM
17	Lymphoma patient with severe immunodepression (serum immunoglobulins < 5 g/l and/or absolute lymphocyte count < 500/mcl) who is candidate to CAR-T therapy	60	Patient with newly diagnosed active MM
18	LLA patient candidate to CAR-T therapy	61	Very old (>80 yrs) patient with active MM ongoing therapy
19	Lymphoma patient soon after CAR-T therapy	62	Patient ongoing therapy for active MM and reporting severe immunodepression (serum immunoglobuline levels <5 g/l)

20	Lymphoma patient aged >60 years soon after CAR-T therapy	63	Patient ongoing therapy for active MM and reporting recurrent infections
21	Lymphoma patient with relevant comorbidities soon after CAR-T therapy	64	Patient ongoing therapy for active MM and reporting relevant comorbidity
22	Lymphoma patient with severe immunodepression (serum immunoglobulins < 5 g/l and/or absolute lymphocyte count < 500/mcl) soon after CAR-T therapy	65	Patient ongoing therapy for previously active MM (currently in complete response)
23	LLA patient soon (< 3 months) after CAR-T therapy	66	Patient previously being treated for MM (currently in complete response)
24	AML patient who achieved CR1 and is candidate to allogeneic SCT	67	Patient with newly diagnosed AML
25	AML patient who achieved CR2 and is candidate to allogeneic SCT	68	Patient ongoing intensive chemotherapy for AML
26	AML patient aged >60 years of age who is candidate to allogeneic SCT	69	Patient ongoing demethylating therapy for AML (without venetoclax)
27	AML patient aged > 60 years of age and with ANC<1000/mcl who is candidate to allogeneic SCT	70	Patient receiving venetoclax-based therapy for AML
28	AML patient aged > 60 years of age and with ANC>=1000/mcl who is candidate to allogeneic SCT	71	Patient receiving oral target therapy for AML
29	AML patient aged > 60 years and reporting ANC< 1000/mcl early after (< 12 months) allogeneic SCT	72	Patient with high-risk MDS ongoing intensive chemotherapy
30	AML patient aged > 60 years and reporting ANC >=1000/mcl early after (< 12 months) allogeneic SCT	73	Patient with high-risk MDS receiving demethylating therapy
31	AML patient aged < 60 years and reporting ANC< 1000/mcl early after (< 12 months) allogeneic SCT	74	Low-risk MDS not receiving immunosuppressive therapy or lenalidomide
32	AML patient aged > 60 years and reporting ALC< 500/mcl early after (< 12 months) allogeneic SCT	75	Low-risk MDS reporting neutrophil count below 1000/mcl
33	AML patient aged > 60 years and reporting ALC >= 500/mcl early after (< 12 months) allogeneic SCT	76	Low-risk MDS ongoing lenalidomide
34	AML patient aged < 60 years and reporting ALC< 500/mcl early after (< 12 months) allogeneic SCT	77	Low-risk MDS ongoing immunosuppressive therapy
35	Treatment-naiive CLL patient	78	CML patient on TKI therapy
36	CLL patient aged > 80 years and on active therapy	79	MPN patient on JAK2-inhibitor therapy
37	CLL patient with relevant comorbidity burden and on active therapy	80	MPN patient on cytoreductive therapy different from JAK2-inhibitor
38	CLL patient with severe immunodepression (immunoglobulin levels < 5 g/) and on active therapy without IGIV support	81	MPN patient off cytoreductive therapy
39	CLL patient with severe immunodepression (immunoglobulin levels < 5 g/) and on active therapy with IGIV support		
40	CLL patient on active therapy and IGIV reporting recurrent infections		

41	CLL patient on active therapy without IGIV support and reporting recurrent infections		
42	CLL patient on BTK-inhibitor therapy and IGIV		
43	CLL patient on BTK-inhibitor therapy and not on IGIV		

Legend to Supplementary Table 5. SCT = stem cell transplant; MM = multiple myeloma; LLA = acute lymphoblastic leukemia; AML = acute myeloid leukemia; CLL = chronic lymphocytic leukemia; IGIV = intravenous immunoglobulins, MDS = myelodysplastic syndrome; CML = chronic myeloid leukemia; TKI = tyrosine kinase inhibitor; ANC = absolute neutrophil count; ALC = absolute lymphocyte count

References

- Abodunrin OR, Olagunju MT, Huang X, Wang J, Hu Z, Shen C. Regional risk factors associated with a dverse outcomes of COVID-19 infection among the older adults: a systematic review and meta-analysis. J Infect Public Health 2025; 18: 102632
- Ahmad O, Weller JF, Kröger N, Wagner Drouet E, Nachbaur D, Steiner N, Teschner D, Kraus S, Bug G, Ajib S, et al. Impact of Allogeneic Hematopoietic Cell Transplantation Post-Sars-Cov-2 Infection: A Retrospective Analysis By the German Cooperative Transplant Study Group. Blood 2024; 144 (Supplement 1): 3554.
- Akbarzadeh MA, Vaez-Gharamaleki Y, Jahanshahlou F, Bavil AG, Hamzehzadeh S, Seifimansour S, Rahimi-Mamaghani A, Hosseini MS.. Outcomes of COVID-19 infection in patients with chronic lymphocytic leukemia: a systematic review and meta-analysis. Rev Assoc. Med Brasil 2024;70: e20240322
- Ali EA, Al-Sadi A, Al-Maharmeh Q, Subahi EA, Bellamkonda A, Kalavar M, Panigrahi K, Alshurafa A, Yassin MA.. SARS_CoV-2 and chronic myeloid leukemia: a systematic review. Front Med 2023;10: 1280271
- Alinaghi S, Karimi A, Barzegary A, Moideganlou H, Vahedi F, Mirghaderi SP, et al. COVID-19 mortality in patients with immunodeficiency and its prdictors: a systematic review. Eur J Med Res 2022;27 : article number 195
- Al-Obaidi MM, Gungor AB, Kurtin SE, Mathias AE, Tanriover B, Zangeneh TT. The Prevention of COVID-19 in High-Risk Patients Using Tixagevimab-Cilgavimab (Evusheld): Real-World Experience at a Large Academic Center. Am J Med. 2023 ;136:96-99.
- Alsoubani M, Chow J. Navigating Coronavirus Disease 2019 in Immunocompromised Populations: Evolving Risk Factors, Treatment, and Outcomes. Infect Dis Clin North Am. 2025 ;39:309-329.
- Anand ST, Vo AD, La J, Do NV, Fillmore NR, Brophy M, Branch-Elliman W, Monach PA. Severe COVID-19 in vaccinated adults with hematologic cancers in the Veterans Health Administration. JAMA Network Open 2024;7: e240288
- Angotzi F, Petrella M, Berno T, Binotto G, Bonetto G, Branca A, Carraro M, Cavaretta CA, Cellini A, D'Amore F, et al. Tixagevimab/Cilgavimab as pre-exposure prophylaxis against SARS-CoV-2 in patients with hematological malignancies. Front Oncol. 2023 ;13:1212752.
- Aparicio C, Willis ZI, Nakamura MM, Wolf J, Little C, Maron GM, Sue PK, Anosike BI, Miller C, Bio LL, et al. Risk Factors for Pediatric Critical COVID-19: A Systematic Review and Meta-Analysis. J Pediatric Infect Dis Soc. 2024;13:352-362.
- Azuly H, Shafat T, Grupel D, Porges T, Abuhasira R, Belkin A, Deri O, Oster Y, Zahran S, Horwitz E, et al; European Society of Clinical Microbiology and Infectious Diseases (ESCMID) Study Group for Respiratory Viruses (ESGREV). Preventing Severe COVID-19 with

Tixagevimab-Cilgavimab in Hematological Patients Treated with Anti-CD20 Monoclonal Antibodies: An International Multicenter Study. Infect Dis Ther. 2025;14:167-180.

- Baek DW, Song GY, Lee HS, Do YR, Lee JH, Yhim HY, Moon JH, Yang DH.. Clinical efficacy of prophylactic intravenous immunoglobulin for elderly DLBCL patients with hypogammaglobulinemia in the COVID-19 pandemic era. Front Oncol 2024:14: 1380492
- Barnes E, Goodyear CS, Willicombe M, Gaskell C, Siebert S, I de Silva T, Murray SM, Rea D, Snowden JA, Carroll M,, et al. SARS-CoV-2 specific immune responses and clinical outcomes after COVID-19 vaccination in patients with immune-suppressive disease. Nature Med 2023;29:1760-74.
- Beer S, Martac I, Schenk A, et al. SARS-CoV-2 immune responses in patients with multiple myeloma under lenalidomide maintenance therapy compared to healthy individuals. Oncol Res Treat 2024;27 (suppl 2): 167-
- Benjamini O, Tadmor T, Avigdor A, Gershon R, Kliker L, Fares F, Atari N, Laevsky I, Abdelkader B, Hod T, Golan-Shany O, Mandelboim M, Rahav G. Efficacy of Preexposure Prophylaxis with Monoclonal Antibody Tixagevimab-Cilgavimab against Emerging SARS-CoV-2 Resistant Variants in Patients with Chronic Lymphocytic Leukemia. Acta Haematol. 2024;147:634-645.
- Bhella SD, Wilkin AM, Hueniken K, Vijenthira A, Sebag M, Wang P, Hicks, LK, Hay AE, McCurdy A, Hosseini-Moghaddam S, ET AL. COVID-19 Humoral Immunity Improves with Third and Subsequent Vaccine Doses in Patients with Plasma Cell Dyscrasias, Particularly in Those Receiving antiCD38 Therapy. Blood, 2024; 144 (Supplement 1): 286
- Bianchi FP, Stefanizzi P, Rizzi D, et al. Burden of COVID-19 disease and vaccine coverages in Apulian splenectomized patients. A retrospective observational study. Br J Haematol 2023;201:1072-80.
- Blixt L, Hedberg P, Eketorp S, Killander Möller I, Lindahl H, Kahn F, Nilsdotter-Augustinsson A, Fredrikson M, Nyström S, Rosenquist R, et al. Severity of COVID-19 in Individuals with Chronic Lymphocytic Leukemia throughout the Pandemic in Sweden: A Nationwide Multiple Register Cohort Study Conducted from 2020 to 2023. Blood 2024; 144 (Supplement 1): 4634
- Bojesen AB, Lund A, Mortesen FV, Kikegard J. Splenectomy and risk of COVID-19 infection, hospitalization, and death. Infect Dis 2021;9:678-83
- Bozkurt C, Hazar V, Malbora B, Küpesiz A, Aygüneş U, Fışgın T, Karakükçü M, Kuşkonmaz B, Kılıç SÇ, Bayırlı D,, et al. COVID-19 disease in children and adolescents following allogeneic hematopoietic stem cell transplantation: a report from the Turkish pediatric bone marrow transplantation study group. Ped Transplant 2024;18: e14758
- Braitsch K, Jeske SD, Stroh J, Hefter M, Platen L, Bachmann Q, Renders L, Protzer U, Götze KS, Herhaus P, et al. Tixagevimab/Cilgavimab for COVID-19 Pre-Exposure Prophylaxis in Hematologic Patients-A Tailored Approach Based on SARS-CoV-2 Vaccine Response. Vaccines (Basel). 2024;12:871.
- Breccia M, Abruzzese E, Accurso V, Attolico I, Barulli S, Bergamaschi M, Binotto G, Bocchia M, Bonifacio M, Caocci G,, et al. COVID-19 infection in chronic myeloid leukeaemia after one year of the pandemic in Italy. A Campus CML report. Br J Haematol 2022:196:559-65

- Bronstein Y, Gat R, Levi S, Cohen YC, Luttwak E, Benyamini N, Shragai T, Vitkon R, Neaman M, Eilaty N, et al. COVID-19 in patients with lymphoproliferative diseases during the Omicron variant surge. Cancer Cell. 2022 ;40:578-580.
- Callegari C, Lazzarotto D, Soravia A, Mutti M, Lauzzana P, Peghin M, Cordella S, Fanin R, Candoni A. Reduced prophylactic effect of tixagevimab-cilgavimab in patients with hematological malignancies and without antibody response after SARS-COV-2 vaccination. Eur J Haematol. 2023;111:668-670.
- Carrillo De Albornoz S, Higgins A, Petrie D, Raje N., Anderson K., Einsele H., Efebera Y., Gay F., Hammond S.P., Lesokhin A.M., et al. Monitoring, prophylaxis, and treatment of infections in patients with MM receiving bispecific antibody therapy: consensus recommendations from an expert panel Blood Cancer Journal 2023 13:1 Article Number 116
- Chai S, Li Y, Li X, Tan J, Abdelrahim MEA, Xu X. Effect of age of COVID-19 inpatients on the severity of the disease: a meta-analysis. Int J Clin Pract 2021;75(19): e14640
- Chakraborty C, Bhattacharya M, Abdelhameed AS. Recent SARS-CoV-2 evolution trajectories indicate the emergence of Omicron's several subvariants and the current rise of KP.3.1.1 and XEC. Virology. 2025;607:110508.
- Chang A, Koff JL, Lai L, Orellana-Noia VM, Surati M, Leal AMK, Ellis ML, Wali B, Moreno A, Linderman SL, et al. Low neutralizing activity of AZD7442 against current SARS-CoV-2 Omicron variants in patients with B-cell malignancies. Blood Adv. 2023;7:2459-2462.
- Chen B, Haste N, Binkin N, Law N, Horton LE, Yam N, Chen V, Abeles S. Real world effectiveness of tixagevimab/cilgavimab (Evusheld) in the Omicron era. PLoS One. 2023;18:e0275356.
- Chen Y, Liu J, Shao S, Song Z, Ma Y, Tuo Y, Fang L, Xu Y, Xu B, Gu W, et al. Characteristics and outcomes of COVID-19 in Chinese immune thrombocytopenia patients: a prospective cohort study. Br J Haematol 2024;204:1207-18
- Chue WX, Lienardi LA, Tan TQW, Kadir IA, Lam JCM. Immune thrombocytopenia exacerbation post COVID-19 vaccination: a systematic review and meta-analysis. Ann Blood 2024;9:23
- Chuleerarux N, Manothummetha K, Moonla C, Sanguankeo A, Kates OS, Hirankarn N, Phongkhun K, Thanakitcharu J, Leksuwankun S, Meejun T,, et al. Immunogencity of SARS_CoV-2 vaccinez in patients with multipe myeloma: a systematic review and meta-analysis. Blood Adv 2022;6:6198-6207
- Copland E, Hirst J, Mi E, Patone M, Chen D, Coupland C, Hippisley-Cox J. Effectiveness and safety of COVID-19 vaccination in people with blood cancer. Eur J Cancer. 2024 ;201:113603.
- Davis JA, Granger K, Roubal K, Smith D, Gaffney KJ, McGann M, Cendagorta A, Thurlapati A, Herbst A, Hendrickson L, et al. Efficacy of tixagevimab-cilgavimab in preventing SARS-CoV-2 for patients with B-cell malignancies. Blood. 2023 ;141:200-203.
- Dawudi Y, Azoyan L, Bonjour M, Steichen O. COVID-19 outcomes among patients with sickle cell disease or risckle cell trait compared to the general population: a systematic review and meta-analyses. Ann Hematol 2024;103: 5071-83

- Demel I, Skopal D, Šafránková E, Rozsívalová P, Jindra P, Šrámek J, Turková A, Vydra J, Labská K, Vedrová J, et al. Effectiveness of tixagevimab/cilgavimab in patients with hematological malignancies as a pre-exposure prophylaxis to prevent severe COVID-19: a Czech retrospective multicenter study. Ann Hematol. 2024;103:981-992.
- Dimbleby B, Greenway W, Burns SO, Richter AG, Shields AM. Health Care Utilisation in a Cohort of Patients with Primary and Secondary Antibody Deficiency in the United Kingdom. J Clin Immunol. 2024 ;45:18.
- Duminuco A, Nardo A, Orofino A, Giunta G, Conticello C, Del Fabro V, Chiarenza A, Parisi MS, Figuera A, Leotta S, et al. Efficacy and safety of tixagevimab-cilgavimab versus SARS-CoV-2 breakthrough infection in the hematological conditions. Cancer. 2024 ;130:41-50.
- Duminuco A, Romano A, Leotta D, La Spina E, Cambria D, Bulla A, Del Fabro V, Tibullo D, Giallongo C, Palumbo GA, et al. Clinical outcome of SARS-CoV-2 infections occurring in multiple myeloma patients after vaccination and prophylaxis with tixagevimab/cilgavimab. Front Oncol. 2023;13:1157610.
- El-Ashwah S, Salamanton-Garcìa J, Bilgin YM, et al. the mortality of COVID-19 in CML patients from 2020 until 2022: results from the EPICOVIDEHA survey. Leuk Lymphoma 2024:65:199-208
- Fares AA, Martinez D, Musleh Ud Din S, Wilkinson R, Chavez JC, Madueno FMV, Sandoval-Sus J. Incidence of infections associated with the use of bispecific antibodies in B-cell non-Hodgkin lymphomas. Blood 2023;142(suppl 1): 2329-
- Farina F, Ferla V, Canziani L, et al. High rate of respiratory tract infections after COVID19 waves in transplant eligible multiple myeloma patients treated with Daratumumab. Haematologica 2024; 109(suppl 2): 29-
- Fatemi B, Rezaei S, Peikanpour M, Dastan F, Saffaei A. Efficacy of intravenous immunoglobulins (IGIV) in COVID_19 patients: a systematic review and meta-analysis. Res Pharm Sci 2023;1:346-357
- Ferhanoglu B, Guilbas Z, Uzay A, et al. Glofitamab in relapsed/refractory diffuse large B cell lymphoma: real world data. Blood 2022; 140(suppl 1): 6704-5
- Frączkiewicz J, Pawińska-Wąsikowska K, Szymbor K, Balwierz W, Skoczeń S, Czyżewski K, Kołtan S, Styczyński J, Małecka A, Irga-Jaworska N, et al. Pre-Exposure Prophylaxis and Treatment with Tixagevimab/Cilgavimab for COVID-19 among Immunocompromised Pediatric Patients. J Clin Med. 2024;13(7):2029.
- Fu H, Cai X, Cui L, Nong W, Li W, Mei H, Yang T, Yue H, Huang Q, An Z, et al. The evolution of preexisting primary immune thrombocytopenia after COVID-19 onset: a nationally representative, prospective, multicentre, observational study. Ann Hematol 2024;103:1549-59
- Gagelmann N, Passamonti F, Wolschke C, Massoud R, Niederwieser C, Adjallè R, Mora B, Ayuk F, Kroeger N. Antobody response after vaccination against SARS-CoV-2 in adults with hematological malignancies: a systematic review and meta-analysis. Haematologica2022;107:1840-9.

- Galli E, Di Rocco A, Pansini I, Frondizi F, Di Palma M, Metafuni E, Piccirillo N, Bianchi M, Cingolani A, Torelli GF, et al. Impact of SARS-CoV-2 vaccination and passive prophylaxis with tixagevimab/cilgavimab on CAR-T patients: a three-year regional experience from the Italian covid pandemic. Bone Marrow Transplant. 2023;58:1394-1396.
- Galvis MM, Bradshaw D, Farmaha J, Jones K, Singh H, Vashisht A, Sahajpal N, Kolhe R, Cortes J.. CML-384 immunological response to SARS-CoV-2 after infection and/or vaccination among chronic myeloid leukemia patients. A prospective study. Clin Lymphoma Myeloma Leukemia 2022 (suppl 2): S294-5
- Geukens T, Brandão M, Laenen A, Collignon J, Van Marcke C, Louviaux I, Demey W, Van Wambeke S, Schrijvers D, et al. Changes in anticancer treatment plans in patients with solid cancer hospitalized with COVID-19: analysis of the nationwide BSMO-COVID registry providing lessons for the future. ESMO Open. 2022 ;7:100610. .
- Graf I, Herndlhofer S, Kundi M, Greiner G, Sperr M, Hadzijusufovic E, Valent P, Sperr WR. Incidence of symptomatic Covid-19 infections in patients with mastocytosis and chronic myeloid leukemia: A comparison with the general Austrian population. Eur J Haematol. 2023 ;110:67-76.
- Guarnera L, Tiravanti I, Guiducci A, Coppola L, Marinoni M, Nunzi A, Laureana R, Cardillo L, Esposito F, Secchi R, et al. Prophylaxis with Tixagevimab/Cilgavimab in chronic lymphocytic leukaemia, a case control study. Ann Hematol. 2024;103:3831-3833.
- Hall VG, Lim C, Saunders NR, Klimevski E, Nguyen THO, Kedzierski L, Seymour JF, Wadhwa V, Thursky KA, Yong MK, et al. Breakthrough COVID-19 is mild in vaccinated patients with hematological malignancy receiving tixagevimab-cilgavimab as pre-exposure prophylaxis. Leuk Lymphoma. 2023;64:1600-1604.
- Hall VG, Nguyen THO, Allen LF, Rowntree LC, Kedzierski L, Chua BY, Lim C, Saunders NR, Klimevski E, Tennakoon GS, et al. Evolution of Humoral and Cellular Immunity Post-Breakthrough Coronavirus Disease 2019 in Vaccinated Patients With Hematologic Malignancy Receiving Tixagevimab-Cilgavimab. Open Forum Infect Dis. 2023;10:ofad550.
- Haraguchi M, Yamamoto H, Watanabe O, Sakoh T, Ishida K, Ogura S, Katoh-Morishima M, Taya Y, Nishida A, Kaji D, et al. Incidence of breakthrough COVID-19 in patients with hematological disorders who received pre-exposure prophylaxis with tixagevimab-cilgavimab: a retrospective study in Japan. Bone Marrow Transplant. 2023 ;58:1051-1053.
- Harandi H, Fallahtafti P, Karimi A, Hashemi SM, Mahalleh M, Ashouri M, Salehi MA, Hoveidaei A.. Examining the immunological response to COVID-19 in multiple myeloma patients: a systematic review and meta-analysis. BMC geriatrics 2024;24:411-
- Hardy N, Vegivinti CTR, Mehta M, Thurnham J, Mebane A, Pederson JM, Tarchand R, Shivakumar J, Olaniran P, Gadodia R, et al. Mortality of COVID-19 in patients with hematological malignancies versus solid tumors: a systematic literature review and meta-analysis. Clin Exp Med. 2023;23:1945-1959.
- Hijano DR, Ferrolino JA, Swift EG, Michaels CA, Max A, Hayden RT, Wolf J, Dallas RH, Greene WL, Richardson JL, et al. SARS-CoV-2 infection in high-risk children following tixagevimab-cilgavimab (Evusheld) pre-exposure prophylaxis: a single-center observational study. Front Oncol. 2023 Aug 3;13:1229655.

- Hirst J, Mi E, Copland E, Patone M, Coupland C, Hippisley-Cox J. Uptake of COVID-19 vaccination in people with blood cancer: Population-level cohort study of 12 million patients in England. Eur J Cancer. 2023;183:162-170.
- Ito Y, Honda A, Kurokawa M. COVID-19 mRNA vaccine in patients with lymphoid malignancy or anti-CD29 antibody therapy: a systematic review and meta-analysis. Clin Lymphoma Myeloma Leukemia 2022;22:e691-e707
- Jondreville L, D'Aveni M, Labussière-Wallet H, Le Bourgeois A, Villate A, Berceanu A, Bezsera SM, Thiebaut A, Boissard-Simonet M, Legrand M, et al. Pre-exposure prophylaxis with tixagevimab/cilgavimab (AZD7442) prevents severe SARS-CoV-2 infection in recipients of allogeneic hematopoietic stem cell transplantation during the Omicron wave: a multicentric retrospective study of SFGM-TC. J Hematol Oncol. 2022;15:169.
- Kaluzhskaya KV, Polyakov YY, Baryakh EA, et al. Treatment and in-hospital overall survival in hematological patients with grade 4 neutropenia and coronavirus infection. Blood 2021; 138 (suppl 1): 3391-
- Kamboj M, Laracy JC, Usiak S, Babady NE, Yan J, Seo SK. Outcomes of hematologic malignancy patients with SARS-CoV-2 breakthrough infections after tixagevimab-cilgavimab during community transmission of monoclonal antibody resistant variants. J Infect. 2023;87:282-285.
- Kertes J, Shapiro Ben David S, Engel-Zohar N, Rosen K, Hemo B, Kantor A, Adler L, Shamir Stein N, Mizrahi Reuveni M, Shahar A. Association Between AZD7442 (Tixagevimab-Cilgavimab) Administration and Severe Acute Respiratory Syndrome Coronavirus 2 (SARS-CoV-2) Infection, Hospitalization, and Mortality. Clin Infect Dis. 2023 ;76:e126-e132.
- Keyzner A, Azzi J, Jakubowski R, Sinitsyn Y, Tindle S, Shpontak S, Kwon D, Isola L, Iancu-Rubin C. Cryopreservation of Allogeneic Hematopoietic Cell Products During COVID-19 Pandemic: Graft Characterization and Engraftment Outcomes. Transplant Proc. 2023 Oct;55(8):1799-1809.
- Kim Linton, John N. Allan, Andrew S Park, Anthony W Wang, Summer Tran, Alex Mutebi, Zhijie Ding, Quan Chen, Tycel J. Phillips. Trends in All-Cause Mortality Rates in Patients with Follicular Lymphoma in the US before and during the COVID-19 Pandemic: A Retrospective Observational Study. Blood, 2024; 144 (Supplement 1): 1703
- Kow CS, Ramachandram DS, Hasan SS, Thiruchelvam K. Systematic review and meta-analysis of anti-CD20 treatments in patients with COVID-19 : an assessment of severe illness and mortality outcomes. Inflammopharmacology 2023;31(6):3339-3355.
- Kyvsgaard ER, Riley C, Clausen MR, Harsløf M, Heftdal LD, Niemann CU, Grønbaek K, Hutchings M, Husby S. Low mortality from COVID-19 infection in patients with B-cell lymphoma after bispecific CD20XCD3 therapy. Br J Hematol 2024:204:356-360
- Lahmer T, Salmanton-García J, Marchesi F, El-Ashwah S, Nucci M, Besson C, Itri F, Jaksic O, Čolović N, Weinbergerová B, et al. Need for ICU and outcome of critically ill patients with COVID-19 and haematological malignancies: results from the EPICOVIDEHA survey. Infection. 2024 Jun;52(3):1125-1141.
- Langerbeins P, Hallek M COVID-19 in patients with hematologic malignancy. Risk Factors for Pediatric Critical COVID-19: A Systematic Review and Meta-Analysis. Blood 2022; 140:236-52.

- Laracy JC, Yan J, Steiger SN, Tan CA, Cohen N, Robilotti EV, Fender J, Cohen S, Korde N, Lee-Teh M, et al. Predictors of SARS-CoV-2 Omicron breakthrough infection after receipt of AZD7442 (tixagevimab-cilgavimab) for pre-exposure prophylaxis among hematologic malignancy patients. Haematologica. 2023 ;108(11):3058-3067.
- Laroye C., Thilly N., Gauthier M., Luc A., Latger-Cannard V., Eschwege V., Bensoussan D., Pochon C., Campidelli A., Rubio M.-T., et al. A French single-center experience on allogeneic stem cell transplant cryopreservation during severe acute respiratory syndrome coronavirus 2 pandemic. Cytotherapy 2023 25:8 (877-884
- Lee YJ, Kim HK, Kim Y, Park SH, Lim JH, Jung J, Choi YS, Jo JC. Tixagevimab/cilgavimab (AZD7442/Evusheld) prevent from COVID19 in patients with hematologic malignancies under active chemotherapy. Ann Hematol. 2024 Jul;103(7):2533-2539.
- Levin MJ, Ustianowski A, De Wit S, Beavon R, Thissen J, Seegobin S, Dey K, Near KA, Streicher K, Kiazand A, et al. Efficacy, Safety, and Pharmacokinetics of AZD7442 (Tixagevimab/Cilgavimab) for Prevention of Symptomatic COVID-19: 15-Month Final Analysis of the PROVENT and STORM CHASER Trials. Infect Dis Ther. 2024; 13:1253-1268.
- Li X, Xu Z, Wang T, Xu X, Li H, Sun Q, Zhou X, Chen G.. Clinical laboratory characteristics of severe patients with coronavirus disease 2019 (COVID-19): a systematic review and meta-analysis: characteristics of severe patients with COVID-19. Clin Epidemiol Global Health 202;9:184-190
- Liang T, Guo K, Ni P, Duan G, Zhang R. The association of sickle cell disorder with adverse outcomes in COVID-19 patients: a meta-analysis. J Med Virol 2023;95:e23120
- Lim KJC, Quach H. the role if intravenous immunoglobulin (IGIV) in reducing infection risk in multiple myeloma (MM) patients recveiving immune-based therapies: a single center experience. Blood 2023;142 (supp 1): 6671-
- Lim SY, Kim JW, Kim JY, Kang SW, Jang CY, Chang E, Yang JS, Kim KC, Jang HC, Kim DS, et al. The association between antibody responses and prolonged viable severe acute respiratory syndrome Coronavirus 2 shedding in immunocompromised patients: a prospective cohort study. J Infect Dis 2024;229: 1722-7.
- Lim YJ, Ward V, Brown A, Phillips E, Kronsteiner B, Malone T, Jennings D, Healy S, Longet S, James T, et al. Immunogenicity of COVID-19 vaccines in patients with follicular lymphoma receiving frontline chemoimmunotherapy. Br J Haematol 2024;205:440-51.
- Liu X, Gan X, Xu J, Wang Y, Huang J, He X, Li Y, Gong Y, Peng B, Niu T COVID-19 vaccination in splenectomized patients with immune thrombocytopenia – Response. Br J Hematology 2024;2:726-7
- Liu X, Zhang Y, Lu L, Li X, Wu Y, Yang Y, Li T, Cao W.. Benefits of high-dose intravenous immunoglobulin on mortality in patients with severe COVID-19: an updated systematic review and meta-analysis. Front Immunol 2023;14:1116738
- Luttwak E, Noy A, Seshan V, Saltzman LA, Greenberger LM. The efficacy of tixagevimab-cilgavimab prophylaxis against Omicron BA.5 variants in patients with hematological malignancies: insights from the Leukemia and Lymphoma Society Registry. Leuk Lymphoma. 2023 Oct;64(10):1727-1729. doi: 10.1080/10428194.2023.2227749.

- Mahmud S, Hossain MF, Muyeed A, Nazneen S, Haque MA, Mazumder H, Mohsin M.. Risk assessment and clinical implications of COVID-19 in multiple myeloma patients: a systematic review and meta-analysis. PLoS ONE 2024;19:e0308463
- Mai A.S., Lee A.R.Y.B., Tay R.Y.K., Shapiro L., Thakkar A., Halmos B., Grinshpun A., Herishanu Y., Benjamini O., Tadmor T., et al. Booster doses of COVID-19 vaccines for patients with haematological and solid cancer: a systematic review and individual patient data meta-analysis. European Journal of Cancer 2022 172 (65-75)
- Mansour RO, El-Ashwah S, Denewer M. The impact of COVID-19 on acute myeloid leukemia patients undergoing allogeneic stem cell transplantation: a concise review. Blood Res 2023;58_13-19
- Marchesi F, Salmanton-García J, Buquicchio C, Itri F, Besson C, Dávila-Valls J, Martín-Pérez S, Fianchi L, Rahimli L, Tarantini G, et al. Passive pre-exposure immunization by tixagevimab/cilgavimab in patients with hematological malignancy and COVID-19: matched-paired analysis in the EPICOVIDEHA registry. J Hematol Oncol. 2023 Apr 1;16(1):32.
- Maruyama S, Wada D, Inoue A, Kashihara M , Shimazaki J , F Saito F , Ishii K , Nakamori Y, Kuwagata Y. Efficacy of initial combination therapy with anti-SARS-CoV-2 antivirals targeting viral clearance in COVID-19 patients with B-cell lymphoma treated with anti-CD20 antibodies: a retrospective single-centre study in Japan. J Infect Chemotherapy 2025;31: article number 102726
- Maruyama S, Wada D, Kanayama S, Shimazu H , Miyano Y , Inoue A , Kashihara M , Okuda K , Saito F , Nakamori Y et al. The evaluation of risk factors for prolonged viral shedding during anti-SARS-CoV-2 monoclonal antibodies and long-term administration of antivirals in COVID-19 patients with B-cell lymphoma treated by anti-CD20 antibody. BMC Infect Dis 2024;24:article number 715
- Mauro FR, Giannarelli D, Galluzzo CM, Visentin A, Frustaci AM, Sportoletti P, Vitale C, Reda G, Gentile M, Levato L, et al. Antibody Response to the SARS-CoV-2 Vaccine and COVID-19 Vulnerability during the Omicron Pandemic in Patients with CLL: Two-Year Follow-Up of a Multicenter Study. Cancers (Basel). 2023 May 30;15(11):2993.
- Mauro FR, Visentin A, Giannarelli D, Molinari MC, Proietti G, Petrella M, Angotzi F, Pepe S, Trentin L, Baroncelli S, et al. Pre-exposure prophylaxis with ixagevimab/cilgavimab in patients with chronic lymphocytic leukaemia treated with targeted agents. Br J Haematol. 2023 May;201(3):564-567
- Meejun T, Srisurapanont K, Manothummetha K, Thongkam A, Mejun N, Chuleerarux N, Sanguankeo A, Phongkhun K, Leksuwankun S, Thanakitcharu J,, et al. Attenuated immunogenicity of SARS-CoV-2 vaccines and risk factors in stem cell transplant recipients: a meta-analysis. Blood Adv 2023;7(18):5624-36.
- Michelon I, Vilbert M, Pinheiro IS, Costa IL, Lorea CF, Castonguay M, Tran TH, Forté S.. COVID-19 outcomes in patients with sickle cell disease and sickle cell trait compared with individuals without sickle cell disease or trait: a systematic review and meta-analysis. eClinicalMedicine 2023;66: article number 102330
- Minoia C, Diella L, Perrone T, Loseto G, Pelligrino C, Attolico I, Pasciolla C, Totaro V, De Candia MS, Spada V et al. Oral anti-viral therapy for earty COVID-19 infection in patients with haematological malignancies: a multicentre prospective cohort. Br J Haematol 2023;202:928-36.

- Mitra AK, Mukherjee UK, Mazumder S, Madhira V, Bergquist T, Shao YR, Liu F, Song Q, Su J, Kumar S,, et al. Sample average treatment effect of the treated (SATT) analysis using counterfactual explanation identifies BMT and SARS-CoV-2 vaccination as protective risk factors associated with COVID-19 severity and survival in patients with multiple myeloma. Blood Cancer J 2023;13: article number 180.
- Moon R, Tien A, Chung J, Pinnelas R, Lee R, Hwang J, Brasfield F, Sahota A. Safety and Efficacy of Intramuscular Tixagevimab-Cilgavimab in Prevention of COVID-19 in Patients Who Are Immunocompromised. Perm J. 2023 Dec 15;27(4):44-54. doi: 10.7812/TPP/22.180.
- Mulanovich P, Chemaly RF, Granwehr B, McConn K, Assistant P, Patel N, Assistant P, Raad II, Adachi J. COVID-19 in a comprehensive cancer center: 2020-2022. Open Forum Infectious Diseases 2022 9 Supplement 2 (S461-)
- Musto P, Salmanton-García J, Sgherza N, Bergantim R, Farina F, Glenthøj A, Cengiz Seval G, Weinbergerová B, Bonuomo V, Bilgin YM, et al. Survival in multiple myeloma and SARS_COV-2 infection through the COVID_19 pandemic: results from the EPICOVIDEHA registry. Hematol Oncol 2024;42(1): e3240
- Naimi A, Yashmi I, Jebeleh R, Imani Mofrad M, Azimian Abhar S, Jannesar Y, Heidary M, Pakzad R. Comorbidities and mortality rate in COVID-19 patients with hematological malignancies: a systematic review and meta-analysis. J Clin Lab Analysis 2022;36(5): article number e24387
- Najjar-Debbiny R, Gronich N, Weber G, Stein N, Saliba W. Effectiveness of Evusheld in Immunocompromised Patients: Propensity Score-Matched Analysis. Clin Infect Dis. 2023 Mar 21;76(6):1067-1073. doi: 10.1093/cid/ciac855.
- Nguyen Y, Flahault A, Chavarot N, Melenotte C, Cheminant M, Deschamps P, Carlier N, Lafont E, Thomas M, Flamarion E, et al. Pre-exposure prophylaxis with tixagevimab and cilgavimab (Evusheld) for COVID-19 among 1112 severely immunocompromised patients. Clin Microbiol Infect. 2022 Dec;28(12):1654.e1-1654.e4. doi: 10.1016/j.cmi.2022.07.015.
- Nooka AK, Rodriguez C, Mateos MV, Manier S, Chastain K, Banerjee A, Kobos R, Qi K, Verona R, Doyle M et al. Incidence, timing, and management of infections in patients receiving teclistamab for the treatment of relapsed/refractory multiple myeloma in the MajesTEC-1 study. Cancer 2024;130:886-900
- Noori M., Azizi S., Abbasi Varaki F., Nejadghaderi S.A., Bashash D. A systematic review and meta-analysis of immune response against first and second doses of SARS-CoV-2 vaccines in adult patients with hematological malignancies. International Immunopharmacology 2022 110 Article Number 109046
- Ntanasis-Stathopoulos I, Filippatos C, Gavriatopoulou M, Malandrakis P, Eleutherakis-Papaiakovou E, Spiliopoulou V, Syrigou RE, Theodorakakou F, Fotiou D, Migkou M, et al. Tixagevimab/Cilgavimab as Pre-Exposure Prophylaxis against COVID-19 for Multiple Myeloma Patients: A Prospective Study in the Omicron Era. Diseases. 2023 Sep 18;11(3):123.
- Oliva A, Cogliati Dezza F, Petrucci F, Romani FE, Morviducci M, Mirabelli FM, Cancelli F, Valeriani E, Marcelli G, Pugliese F, et al. Outcome of COVID-19 patients with haematological malignancies after the introduction of vaccination and monoclonal antibodies: results from the HM-COV 2.0 study. Clin Exp Med. 2023 Oct;23(6):2275-2285.

- Ono R, Kitagawa I. SARS-CoV-2 infection-induced immune thrombocytopenia: a systematic review of current reports. Ann Hematol 2024:103:3921-39
- Otiniano A, van de Wyngaert Z, Brissot E, Dulery R, Gozlan J, Daguenel A, Abi Aad Y, Ricard L, Stocker N, Banet A Tixagevimab/cilgavimab for Omicron SARS-CoV-2 infection in patients with haematologic diseases. Bone Marrow Transpl 2023; 58:340–342
- Pagano L, Salmanton-García J, Marchesi F, Blennow O, Gomes da Silva M, Glenthøj A, van Doesum J, Bilgin YM, López-García A, Itri F., et al. Breakthrough COVID-19 in vaccinated patients with hematologic malignancies: results from the EPICOVIDEHA survey. Blood 2022 140:26 (2773-2787)
- Pagano L., Salmanton-Garcia J., Marchesi F., Lopez-Garcia A., Lamure S., Itri F., Gomes-Silva M., Dragonetti G., Falces-Romero I., van Doesum J., et al. COVID-19 in vaccinated adult patients with hematological malignancies: preliminary results from EPICOVIDEHA. Blood. 2022;139:1588–1592.
- Passamonti F, Cattaneo C, Arcaini L, Bruna R, Cavo M, Merli F, Angelucci E, Krampera M, Cairoli R, Della Porta MG, et al; ITA-HEMA-COV Investigators. Clinical characteristics and risk factors associated with COVID-19 severity in patients with haematological malignancies in Italy: a retrospective, multicentre, cohort study. Lancet Haematol. 2020 Oct;7(10):e737-e745.
- Perry C, Luttak E, Balaban R, Efficacy of the BNT162b2 mRNA COVID-19 vaccine in patients with B-cell non-Hodgkin lymphoma. Blood Adv 2021;5:3053-61
- Pike A, Fox N, McKinley CE, et al. SARS-CoV-2 antibody and T-cell responses in patients with paroxysmal nocturnal hemoglobinuria and plastic anemia after four COVID-19 vaccinations. Blood 2023; 142 (suppl 2): 2712-
- Polyakov Y, Kaluzhskaya K, Baryakh E, Gemdzhian E, Misyurina E, Zhelnova E, Yatskov K, Kochneva O, Andreev S, Chudnova T,, et al. Therapy and hospital mortality predictors in patients with lymphoproliferative disorders and concomitant COVID-19 infection. HemaSphere 2022;6 8suppl 3): 2811-2812
- Radich JP, Kok CH, Chelysheva EY, Cortes JE, Jiang Q, Mauro M, Milojcovic D, Moiraghi B, Nicolini FE, Ongondi M et al. A risk model for CML patients with COVID-19: importance of molecular response in the context of age, comorbidities and country income. Blood 2022;140(suppl1):9617-19
- Rahul Ramaswamy, Laith Fakhoury, Peter Zhao, Ningying Wu, Feng Gao, Geoffrey L. Uy, John F. DiPersio, Zachary D. Crees. Waning Long Term Efficacy of COVID-19 Post-Transplant Vaccination for Prevention of COVID-19 Adverse Events in Transplant and Cellular Therapy (TCT) Patients. Blood, 2024; 144 (Supplement 1): 3532.
- Randi BA, Higashino HR, da Silva VP, Xavier EM, Rocha V, Costa SF. COVID-19 in hematopoietic stem-cell transplant recipients: a systematic review and meta-analysis of clinical characteristics and outcomes. Rev Med Virol 2023; 33: e2483

- Reimann P, Petzer V, Mündlein A, Hartmann B, Severgnini L, Winkler A, Lang T, Huynh M, Gasser K, Rüger J, et al. Efficacy and safety of tixagevimab/cilgavimab as passive immunisation against COVID-19 infections in patients with hematological malignancies. Ann Hematol. 2024 Jun;103(6):2123-2131.
- Reynolds GK, Maclean M, Cliff ERS, Teh BW, Thursky KA, Slavin MA, Anderson MA, Hawkes EA.. Infections in lymphoma patients treated with bispecific therapies: a systematic review and meta-analysis. Blood 2023;142: 6896-
- Rigolin GM, Urso A , Soddu S , Moia R, Dondolin R, Olivieri A, Scortechini I, Frustaci A, Deodato M, Daghia G et al. Severe COVID-19 Infection in Patients with Chronic Lymphocytic Leukemia or Indolent B-Cell Non-Hodgkin Lymphoma Who Received Pre-Exposure Prophylaxis with Tixagevimab and Cilgavimab in Italy: Preliminary Results of the Observational Study By the Gimema Working Party on Chronic Lymphoproliferative Disorders and By the Fondazione Italiana Linfomi. Blood 2024, 144: 1872-1873
- Rivière E. British Society for Haematology guidelines to improve the care of asplenic patients: much work done, some remaining and a call for national registries. Br J Haematol 2024; 204:1573-6
- Rossi G, Salmanton-García J, Cattaneo C, Marchesi F, Dávila-Valls J, Martín-Pérez S, Itri F, López-García A, Glenthøj A, Gomes da Silva M, et al. Age, successive waves, immunization, and mortality in elderly COVID-19 hematological patients: EPICOVIDEHA findings. Int J Infect Dis 2023; 137: 98-130.
- Rotterdam J, Thiaucourt M, Weiss C, Schwaab J, Reiter A, Kreil S, Steiner L, Fenchel S, Popp HD, Hofmann WK,, et al. Definition of factors associated with negative antibody response after COVID-19 vaccination in patients with hematological diseases. Ann Hematol 2022:101:1825-34
- Sakuraba A, Luna A, Micic D. Serologic response following SARS-COV2 vaccination in patients with cancer: a systematic review and meta-analysis. J Hematol Oncol. 2022 Feb 5;15(1):15.
- Salmanton-García J, Marchesi F, Farina F, Weinbergerová B, Itri F, Dávila-Valls J, Martín-Pérez S, Glenthøj A, Hersby DS, Gomes da Silva M, et al; EPICOVIDEHA registry. Decoding the historical tale: COVID-19 impact on haematological malignancy patients-EPICOVIDEHA insights from 2020 to 2022. EClinicalMedicine. 2024 Mar 18;71:102553.
- Sanchez-Pina JM, Rodríguez Rodriguez M, Castro Quismondo N, Gil Manso R, Colmenares R, Gil Alos D, Paciello ML, Zafra D, Garcia-Sanchez C, Villegas C. Clinical course and risk factors for mortality from COVID-19 in patients with haematological malignancies. Eur J Haematol 2020; 105:597–607
- Sarkar S, Khanna P, Singh AK. The impact of neutrophil-lymphocyte count ratio in COVID-19: a systematic review and meta-analysis. J Intensive Care Med 2022;37/7): 857-69.
- Silva Borborema T, Moreira Brito JC, Lima Batista EM, Siqueira Batista R. Case fatality rate and severity of COVID-19 among patients with sickle cell disease: a systematic review and meta-analysis. Hemoglobin 2023;47:85-96

- Siniaev A, Chebykina DA, Popova MO, Rogacheva JA, Zorina NA, Minaeva NV, Voloshin SV, Bondarenko SN, Moiseev IS, Kulagin AD. Tixagevimab/cilgavimab prophylaxyis against SARS-CoV-2 in patients with hematological malignancies. Cell Ther Transplant 2023;12:58-61
- Siniaev A, Popova M, Rogacheva Y, Ianbukhtina V, Kulagin E, Vladovskaya M, Bondarenko S, Moiseev I, Kulagin AD. Impact of prior COVI-19 infection on allogeneic hematopoetic stem cell transplantation outcomes. Clin Transplant 2024;38:e15331
- Sivasubramanian BP, Joshi S, Ravikumar DB, Madhumithaa Jagannathan, Babu S, Sripathi SR, Javvaji A, Jain P, Kumar Shanmugam D, et al. COVID-19 in acute myeloid leukemia (AML) and myelodysplastic syndrome (MDS): a propensity matched analysis (2020-2021). Front. Oncol. 14:1446482.
- Soeroto AY, Yanto TA, Kurniawan A, Hariyanto TI. Efficacy and safety of tixagevimab-cilgavimab as pre-exposure prophylaxis for COVID-19: a systematic review and meta-analysis. Rev Med Virol 2023;33:e2420
- Sood A, Sangari A, Wright J, Locke M, Elegickal J, Hiu L, Aung WY, Singhal A, George R, Nieto MJ. Influence of COVID-19 on Acute Myeloid Leukemia Treatment Efficacy: Insights from a Propensity Score-Matched Cohort Study Blood (2024) 144 (Supplement 1): 1492
- Stahl M, Narendra V, Jee J, Derkach A, Maloy M, Geyer MB, Mato AR, Roeker LE, Tallman MS, Shah GL, et al. Neutropenia in adult acute myeloid leukemia patients represents a powerful risk factor for COVID-19 related mortality. Leuk Lymphoma 2021;62:1940-8
- Stuver R, Shah GL, Korde NS, Roeker LE, Mato AR, Batlevi CL, Chung DJ, Doddi S, Falchi L, Gyurkocza B, et al. Activity of AZD7442 (tixagevimab-cilgavimab) against Omicron SARS-CoV-2 in patients with hematologic malignancies. Cancer Cell. 2022 Jun 13;40(6):590-591.
- Suribahtla R, Starkey T, Ionescu MC, Pagliuca A, Richter A, Lee LYW. Systematic review and meta-analysis of the clinical effectiveness of tixagevimab-cilgavimab for prophylaxis of COVID-19 in immunocompromised patients. Br J Haematol 2023;201:813-23
- Tatetsu H, Higuchi Y, Shichijo T, Oda K, Nakata H, Yasunaga JI, Nosaka K, Matsuoka M. Prophylactic effect of tixagevimab-cilgavimab on COVID-19 infection and death in Japanese patients with B cell lymphoma. Int J Hematol. 2023 Aug;118(2):303-305.
- Terpos E., Musto P., Engelhardt M., Delforge M., Cook G., Gay F., van de Donk N.W.C.J., Ntanasis-Stathopoulos I., Vangsted A.J., Driessen C., et al. Management of patients with multiple myeloma and COVID-19 in the post pandemic era: a consensus paper from the European Myeloma Network (EMN) Leukemia 2023 37:6 (1175-1185)
- Thurlapati A, McGann M, Davis JA, Granger K, Roubal K, Smith D, Gaffney KJ, Cendagorta A, Herbst A, Hendrickson L, Hess BT, Hashmi H. Role of tixagevimab-cilgavimab in preventing SARS-CoV-2 in hematopoietic stem cell transplant and chimeric antigen receptor T-cell therapy recipients. J Med Virol. 2023 Aug;95(8):e29027
- Trepl J, Pasin C, Schneidawind D, Mueller NJ, Manz MG, Bankova AK, Abela IA. Evaluating tixagevimab/cilgavimab prophylaxis in allogeneic haematopoietic cell transplantation recipients for COVID-19 prevention. Br J Haematol. 2024 May;204(5):1908-1912.
- Uaprasert N., Pitakkitnukun P., Tangcheewinsirikul N., Chiasakul T., Rojnuckarin P. Serologic response following SARS-COV2 vaccination in patients with cancer: a systematic review and meta-analysis Blood Cancer Journal 2022 12:12 Article Number 173

- van de Donk NWCJ, Bahlis N, Costa LJ, Mateos MV, Nooka AK, Perrot A, Garfall AL, Thaman P, Qi K, Uhlar C, et al. Impact of COVID-19 on outcomes with teclistamab in patients with relapsed/refractory multiple myeloma in the phase 1/2 MajesTEC-1 study. Blood Cancer J. 2024 ;14(1):186.
- van Doesum JA, Salmanton-García J, Marchesi F, Di Blasi R, Falces-Romero I, Cabirta A, Farina F, Besson C, Weinbergerová B, Van Praet J, et al. Impact of SARS-CoV-2 vaccination and monoclonal antibodies on outcome post-CD19-directed CAR T-cell therapy: an EPICOVIDEHA survey. Blood Adv. 2023 ;7:2645-2655.
- Vegivinti C.T.R., Musunuru T.N., Jitta S.R., Mehta M., Shivkumar J., Thakur R.K., Keesari P.R., Pulakurthi Y.S., Hardy N., Thurnham J., et al. Mortality of COVID-19 in Patients with Hematological Malignancies Versus Solid Tumors: A Systematic Literature Review and Meta-Analysis. Blood 2022 140 Supplement 1 (13218-13219)
- Vergori A, Cozzi Lepri A, Chiuchiarelli M, Mazzotta V, Metafuni E, Matusali G, Siciliano V, Paulicelli J, Alma E, Siniscalchi A, et al. Risk of SARS-CoV-2 infection in patients with hematologic diseases receiving tixagevimab/cilgavimab as pre-exposure prophylaxis in most recent Omicron sublineages era. Int J Infect Dis. 2024 ;144:107042.
- Wang X, Sima L. Antibody response after vaccination against SARS-CoV-2 in adults with hematological malignancies: a systematic review and meta-analysis. J Infect. 2022 :S0163-4453(22)00674-0.
- Wang Y, Zheng J, Zhu K, Xu C, Wang D, Hou M. The effect of tixagevimab-cilgavimab on clinical outcomes in patients with COVID-19: a systematic review with meta-analysis. J Infect 2023;86:e15-17
- Wu X, Xu K, Zhan P, Liu H, Zhang F, Song Y, Lv T. Comparative efficacy and safety of COVID-19 vaccines in phase III trials: a network meta-analysis. BMC Infect Dis 2024;24: article number 234
- Xue E, Scorpio G, Ruggeri A, Clerici D, Farina F, Campodonico E, Acerbis A, Fiore P, Bruno A, Carrabba MG, et al. Impact of tixagevimab/cilgavimab prophylaxis in patients undergoing allogeneic hematopoietic stem cell transplants and CAR T-cell therapy: A single center experience. Curr Res Transl Med. 2023 Jul-Sep;71(3):103402.
- Yang W, Zhang D, Li Z, Zhang K. Predictors of poor serologic response to COVID-19 vaccine in patients with cancer: a systematic review and meta-analysis. Eur J Cancer 2022;172:41-50.
- Yasmeen Choudhri, Olivia Lopes, Caitlyn Vlasschaert, David M. Masloveand Michael J. Rauh. Associations of Lineage-Specific Clonal Hematopoiesis with COVID-19 Hospitalization and Mortality. Blood, 2024, 144 Suppl 1: 1295
- Yigenoglu TN, Ata N, Altuntas F, Bascı S, Dal MS, Korkmaz S, Namdaroglu S, Basturk A, Hacıbekiroglu T, Dogu MH. The outcome of COVID-19 in patients with hematological malignancy. J Med Virol 2021; 93:1099–1104
- Zamprogna G, Frustaci AM, Travi G, Borella C, Reda G, Motta M, Deodato M, Bossi E, Mattiello V, Ferrari MB, et al. Tixagevimab/Cilgavimab Pre-exposure Prophylaxis in Patients With Lymphoproliferative Disorders on BTKi. Hemasphere. 2023;7:e880.

- Zerbit J, Detroit M, Meyer A, Decroocq J, Deau-Fischer B, Deschamps P, Birsen R, Mondesir J, Franchi P, Miekoutima E, et al. Patients with Hematological Malignancies Treated with T-Cell or B-Cell Immunotherapy Remain at High Risk of Severe Forms of COVID-19 in the Omicron Era. Viruses. 2022;14:2377.
- Zhang SX, Arroyo Marioli F, Gao R, Wang S. A Second Wave? What Do People Mean by COVID Waves? - A Working Definition of Epidemic Waves. Risk Manag Healthc Policy. 2021;14:3775-3782.
- Zhao JY, Zhang LL, Kuang ZX, Xu J, Wang WW, Pan H, Gao Z, Li WW, Fang LW, Song Z, Shi J. Evaualtion of the lcinical manifestations of COVID-19 in patients with plastic anemia undergoing immunosuppressive therapy: a prospective cohort study (NICHE). Zhonghua xueyexue zazhi 2023 44:11 (900-905)
- Zhong Z., Wang X., Guo J., Li X., Han Y. Mortality of COVID-19 in patients with hematological malignancies versus solid tumors: a systematic literature review and meta-analysis. Journal of Intensive Care Medicine 2024 39:9 (840-852).
- Zhu H, Lu X, Zhang X, Hua H, Zhang J, Miao Y, Gu W, Xu M, Lu X, Li B, et al. Multi-center study of COVID-19 infection in elderly patients with lymphoma: on behalf of the Jiangsu Cooperative Lymphoma Group (JCLG). Ann Hematol 2024;103:5713-27
- Zhu X., Jiang Q., Lu J., Sun Y., Zhao X., Yang S., Tang F., Yu W., Zhao T., Liu X., et al. COVID-19 infection in patients with haematological malignancies: A single-centre survey in the latest Omicron wave in China. British Journal of Haematology 2023 202:1 (31-39)
- Zhu YJ, Wang JY, Wu CN, Yu BY, Liu TT, Liu Y, Zhang LL. Equity evaluation of intensive care unit admission based on comorbidity in hospitalized patients with COVID-19: a cross-sectional analysis. Front Public Health 2024;12: 1430462-
- Zinellu A, Mangoni AA. A systematic review and meta-analysis of the association between the neutrophil, lymphocyte, and platelet count, neutrophil-to-lymphocyte ratio, and platelet-to-lymphocyte ratio and COVID-19 progression and mortality. Expert Rev Clin Immunol 2022;18:1187-1202
- Marasco V, Piciocchi A, Candoni A, Pagano L, Guidetti A, Musto P, Bruna R, Bocchia M, Visentin A, Turrini M, Tucci A, Pilerci S, Fianchi L, Salvini M, Galimberti S, Coviello E, Selleri C, Luppi M, Crea E, Fazi P, Passamonti F, Corradini P. Neutralizing monoclonal antibodies in haematological patients paucisymptomatic for COVID-19: The GIMEMA EMATO-0321 study. Br J Haematol. 2022 Oct;199(1):54-60.
- Bavaro DF, Diella L, Belati A, Metrangolo G, De Santis L, Spada V, Camporeale M, Dargenio A, Brindicci G, Balena F, Fiordelisi D, Signorile F, Loseto G, Pasciolla C, Minoia C, Attolico I, Perrone T, Simone S, Rendina M, Giovine N, Di Gennaro F, Musto P, Guarini A, Di Leo A, Gesualdo L, Dell'Aera M, Saracino A. Efficacy of Remdesivir and Neutralizing Monoclonal Antibodies in Monotherapy or Combination Therapy in Reducing the Risk of Disease Progression in Elderly or Immunocompromised Hosts Hospitalized for COVID-19: A Single Center Retrospective Study. Viruses. 2023 May 19;15(5):1199.
- Attolico I, Tarantini F, Carluccio P, Schifone CP, Delia M, Gagliardi VP, Perrone T, Gaudio F, Longo C, Giordano A, Sgherza N, Curci P, Rizzi R, Ricco A, Russo Rossi A, Albano F, Larocca AMV, Vimercati L, Tafuri S,Musto P. Serological response following BNT162b2 anti-SARS-CoV-2

mRNA vaccination in haematopoietic stem cell transplantation patients. Br J Haematol. 2022 Feb;196(4):928-931. doi: 10.1111/bjh.17873. Epub 2021 Oct 18. PMID: 34664267; PMCID: PMC8652694.

- Attolico I, Tarantini F, Carluccio P, Musto P. Serological response following anti-SARS-CoV-2 vaccination in hematopoietic stem cell transplantation patients depends upon time from transplant, type of transplant and "booster" dose. Haematologica. 2022 May 1;107(5):1218. doi: 10.3324/haematol.2022.280619. PMID: 35045696; PMCID: PMC9052926.ID:PMC9796521.
